# Bronchopulmonary dysplasia in neonates born to mothers with preeclampsia: Impact of small for gestational age

**DOI:** 10.1371/journal.pone.0204498

**Published:** 2018-09-24

**Authors:** Pauline Dravet-Gounot, Héloïse Torchin, François Goffinet, Marie-Stéphanie Aubelle, Mayass El Ayoubi, Claire Lefevre, Pierre-Henri Jarreau, Elodie Zana-Taïeb

**Affiliations:** 1 Service de Médecine et Réanimation néonatales, Maternité Port-Royal, Assistance Publique–Hôpitaux de Paris, Hôpital Cochin, Paris, France; 2 DHU Risques et grossesse, Maternité Port-Royal, Paris, France; 3 INSERM, U1153, Paris, France; 4 Service de Gynécologie-obstétrique, Maternité Port-Royal, Assistance Publique–Hôpitaux de Paris, Hôpital Cochin, Paris, France; 5 INSERM, U1141, Paris, France; Center of Pediatrics, GERMANY

## Abstract

**Background and objectives:**

Small for gestational age and preeclampsia have both been described as risk factors for bronchopulmonary dysplasia in preterm neonates, but their respective role in the occurrence of bronchopulmonary dysplasia is debated. We evaluated the relation between small for gestational age and bronchopulmonary dysplasia in neonates born to mothers with preeclampsia. We hypothesized that low birth weight is still associated with bronchopulmonary dysplasia in this homogeneous population.

**Methods:**

Retrospective single-center cohort study including 141 neonates born between 24 and 30 weeks’ gestation to mothers with preeclampsia. The main outcome measure was moderate to severe bronchopulmonary dysplasia at 36 weeks’ postmenstrual age. Neonates born small for gestational age (birthweight < 10^th^ percentile on the AUDIPOG curves) were compared to those with appropriate birthweight for gestational age by bivariable analyses and logistic regression models, estimating odds ratios (ORs) and 95% confidence intervals (CIs).

**Results:**

Bronchopulmonary dysplasia rates were 61.5% (32/52) and 27.4% (20/73) for small for gestational age and appropriate birthweight for gestational age neonates (p < .001). On adjustment for gestational age and other confounding factors, the risk of moderate to severe bronchopulmonary dysplasia was greater for small for gestational age than appropriate birthweight for gestational age neonates (adjusted OR = 5.9, 95% CI [2.2–15.4]), as was the composite outcome death or moderate to severe bronchopulmonary dysplasia (adjusted OR = 4.7, 95% CI [1.9–11.3]).

**Conclusions:**

Small for gestational age was associated with bronchopulmonary dysplasia in very preterm neonates born to mothers with preeclampsia.

**Registration number:**

CNIL no. 1747084.

## Introduction

Bronchopulmonary dysplasia (BPD) is the main respiratory sequelae of preterm birth; it is characterized by arrested alveolar development with reduced number but increased size of alveoli and impaired capillaries [1]. Its incidence is stable despite recent advances in prevention and management and is inversely related to birth weight and gestational age (GA) [2]. Numerous antenatal and postnatal factors that can affect BPD development in an immature lung include infections, patent ductus arteriosus (PDA), mechanical ventilation and hyperoxia [3].

Small for GA (SGA) has been described as a risk factor for BPD. Several studies found the risk of BPD two- to six-fold higher for SGA preterm infants than AGA newborns [2,4,5]. One of the main etiologies of SGA is preeclampsia, which is characterized by gestational hypertension and proteinuria and affects 2% to 8% of pregnancies [6]. Preeclampsia as a risk factor for BPD has several hypotheses. First, this pregnancy disease often leads to SGA and premature birth, because delivery is the only treatment. As well, preeclampsia by itself could induce BPD. Indeed, its pathogenesis involves an imbalance between pro- and anti-angiogenic factors [7,8,9], which are shared by the fetus, and could impair the vascular and alveolar development of the fetal lungs [10,11]. Previous studies of BPD incidence in SGA preterm infants did not account for the causes of growth restriction [12,13]. As a result, they mostly compared SGA infants born to mothers with vascular diseases of pregnancy and AGA infants born to normotensive mothers. However, the BPD–SGA relation may be explained wholly or in part by the presence of preeclampsia.

To help disentangle the respective role of preeclampsia and SGA in the development of BPD, we evaluated the impact of SGA on the occurrence of BPD in neonates born to mothers with preeclampsia. We hypothesized that low birth weight is still associated with BPD in this homogeneous population.

## Methods

This retrospective cohort study included preterm infants born between 24 and 30 weeks’ gestation and hospitalized between January 1, 2009 and December 31, 2013 in a level III neonatal intensive care unit at Port Royal Maternity (Cochin hospital, Paris, France). Infants were included if their mothers had preeclampsia (i.e., gestational hypertension [systolic blood pressure ≥ 140 mmHg or diastolic blood pressure ≥ 90 mmHg] occurring after gestational week 20) with proteinuria ≥ 0.3 g/24 h, or eclampsia (i.e., preeclampsia with seizures during pregnancy or shortly after delivery). We excluded mothers with preexisting or gestational hypertension and isolated Hemolysis, Elevated Liver enzymes, Low Platelet count syndrome (HELLP syndrome) not associated with preeclampsia and infants with severe birth defects and chromosomal aberrations. Data were retrospectively collected by using the computer database Perinat Collection, PremUp. The collection of perinatal data was authorized by the National Data Protection Authority (CNIL no. 1747084) and the study was approved by the ethics committee of the French Neonatal Society. The CNIL authorizations allow us to collect and use data retrospectively from patients hospitalized in the unit. There was no consent specifically requested from families but they receive information about clinical research upon admission to the unit and their non-opposition is collected. All data were fully anonymized.

### Outcomes

The primary outcome was moderate to severe BPD defined as oxygen requirement for at least 28 days and persistent need for oxygen or ventilatory support at 36 weeks’ postmenstrual age (PMA) [1]. The secondary outcomes were the composite outcome death or moderate to severe BPD at 36 weeks’ PMA and the main complications of prematurity: in-hospital deaths, necrotizing enterocolitis, neonatal late-onset infections, severe cerebral lesions, PDA, hemodynamic insufficiency requiring vascular filling or inotropic drugs, hyperglycemia requiring insulin treatment, and retinopathy of prematurity.

### Perinatal data

Maternal and neonatal data were collected from medical records. Abnormal Doppler findings during pregnancy mean reduced, absent, or reversed umbilical artery end-diastolic flow; increased middle cerebral artery end-diastolic flow or cerebral redistribution process; reduced, absent, or reversed atrial flow in the ductus venosus; diastolic notch or abnormal pulsatility index on uterine artery. The antenatal corticosteroid course was considered complete if betamethasone was administered twice at 24-h intervals, incomplete if only one injection was administered, or absent. GA was estimated by the first trimester ultrasound if available and otherwise with the last menstrual date. SGA was classified as birth weight below the 10^th^ percentile according to the sex-specific AUDIPOG curves [14] and AGA otherwise. PDA was diagnosed with clinical signs and echocardiographic findings. Necrotizing enterocolitis was diagnosed as Bell’s stage ≥ 2 [15]. Neonatal late-onset infections were defined as any positive culture from blood, cerebrospinal fluid, tracheal aspirate or urine sample occurring more than 72 h after birth. Severe cerebral lesions were considered grade III and IV intraventricular hemorrhage (IVH III-IV) according to the Papile classification and periventricular leukomalacia [16].

Ventilation protocols, fluid intake and PDA management were unchanged throughout the study. High-frequency oscillation ventilation (HFO) was used if hypercarbia or hypoxia persisted despite high pressure with conventional ventilation and in cases of pulmonary hemorrhage. Inhaled corticosteroids were used for extubating infants with continuous assisted-ventilation dependency. Systemic corticosteroids were used in rescue for infants with severe respiratory disease, repeated extubation failure and continuous assisted-ventilation dependency.

### Statistical analysis

Categorical variables are described with number (%) and were compared by chi-square or Fisher exact test. Continuous variables are described with median and interquartile range (IQR) and were compared by Wilcoxon test. We compared the main maternal, obstetrical and neonatal characteristics between SGA and AGA newborns. The associations between SGA and neonatal outcomes were analyzed by bivariable analyses, then adjusted on GA. The associations between SGA and moderate or severe BPD and death or moderate to severe BPD were further adjusted on potential confounding factors selected among characteristics associated with BPD in our sample and relevant factors from the literature. Results from logistic regression models were quantified by odds ratios (ORs) and 95% confidence intervals (95% CIs). Significance was set at p≤.05. Analyses involved use of SAS v9.3 (SAS Inst. Inc., Cary, NC, USA).

## Results

### Prenatal and neonatal characteristics at birth

**Fig 1:**
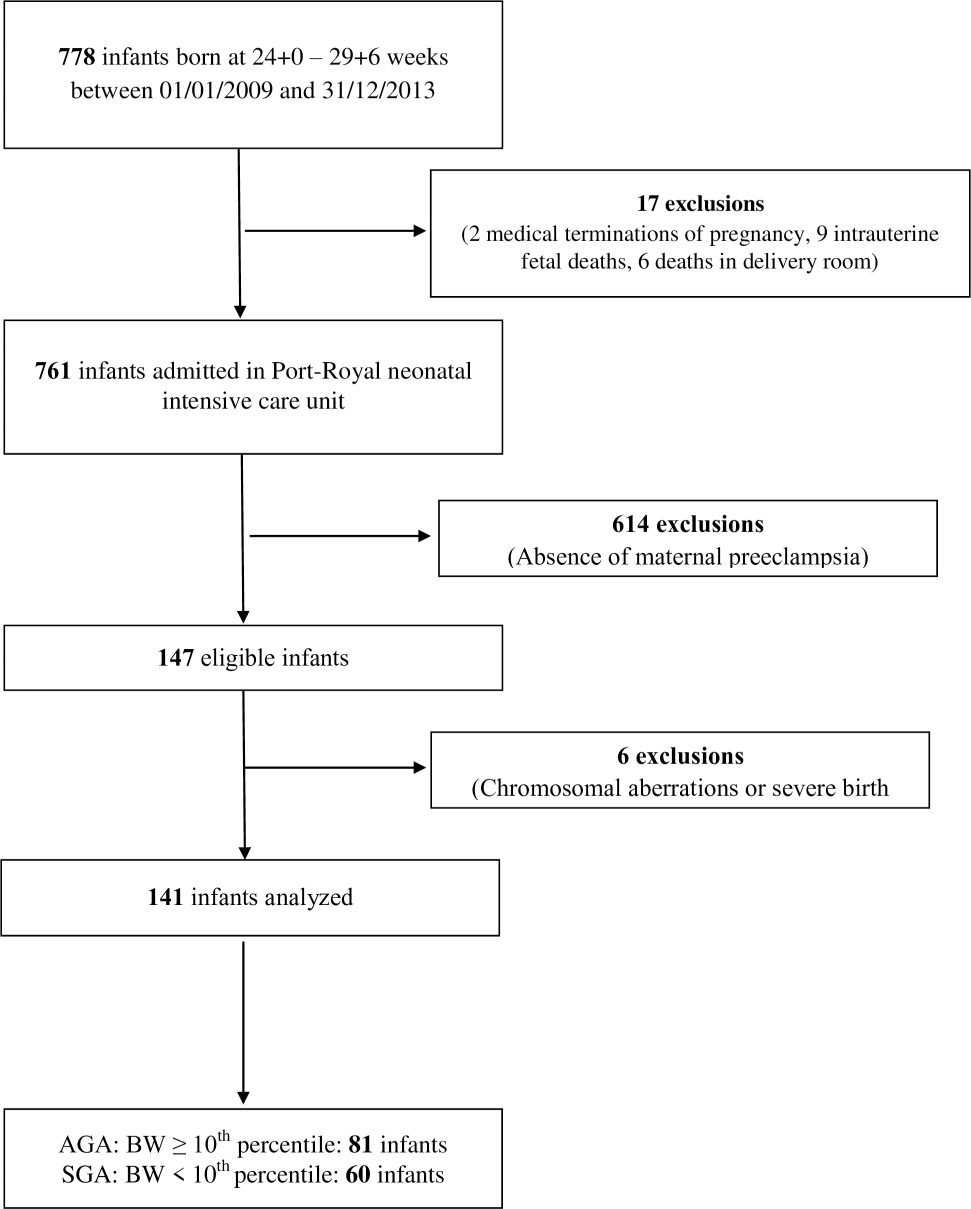
Flow chart of preterm infants in the study.

We screened 778 infants born between 24 and 30 weeks’ gestation and hospitalized between January 1, 2009 and December 31, 2013 (Fig 1). We analyzed data for 141 neonates (81 AGA and 60 SGA) born to mothers with preeclampsia between 2009 and 2013 and hospitalized in the Cochin Port Royal neonatal intensive care unit (Fig 1). SGA and AGA neonates did not differ in prenatal or neonatal characteristics (Table 1) or rates of multiple pregnancies, cesarean-section deliveries and antenatal steroid use. Abnormal Doppler findings during pregnancy were more frequent and GA at birth was lower for SGA than AGA neonates. At birth, lactate level and Clinical Risk Index for Babies (CRIB) score were significantly higher and platelet count was lower for SGA than AGA neonates.

**Table 1:** Prenatal and neonatal characteristics by birth weight of preterm infants: appropriate birthweight for gestational age (GA) and small for GA (SGA).

	AGA (N = 81)		SGA (N = 60)		p
**Multiple pregnancy** (n, %)	8	9.9	5	8.3	.53
**Caesarean section** (n, %)	80	98.8	60	100.0	.99
**Pregnancy complication** (n, %)					
Preeclampsia	80	98.8	59	98.3	.99
Preeclampsia + HELLP syndrome	28	34.6	11	18.3	**.03**
Eclampsia	1	1.2	2	3.3	.57
**Antenatal steroids** (n, %)					.77
None	4	4.9	2	3.3	
Incomplete course	6	7.4	3	5.0	
Complete course	71	87.7	55	91.7	
**Pathological Doppler findings** (n, %)	54	66.7	52	86.7	**< .01**
**GA** (median, IQR)	28.3	[27.3;29.3]	28.0	[27.3;28.5]	**.04**
**Birth weight (g)** (median, IQR)	920	[805;1035]	655	[600;740]	**< .001**
**Birth weight (percentiles)** (median, IQR)	31	[18;46]	3	[1;6]	**< .001**
**Sex: boys** (n, %)	34	42.0	23	38.3	.66
**Cord pH** (median, IQR)	7.28	[7.23;7.31]	7.27	[7.21;7.33]	.57
**Lactate level at birth** (median, IQR)	4.1	[2.8;6.9]	5.1	[3.8;8.5]	**< .01**
**CRIB score** (median, IQR)	3	[1;5]	7	[5;9]	**< .001**
**Platelet count at birth** (x10^3^/mm^3^) (median, IQR)	183	[140;204]	150	[125;176]	**< .01**

IQR: interquartile range. CRIB: Clinical Risk Index for Babies

Any missing data for each variable

### Neonatal clinical course

SGA newborns more frequently required HFO ventilation and final extubation occurred later than did AGA newborns (Table 2). They also had a longer duration of non-invasive ventilation. SGA infants more frequently received inhaled corticosteroids than AGA infants, although not significantly (13.3% vs 4.9%, p = .08). Insulin requirement was twice more frequent for SGA than AGA infants. SGA infants also more frequently needed vascular filling and inotropic drugs as well as red blood cell transfusions, and the platelet count nadir was significantly lower than AGA newborns.

**Table 2:** Main respiratory, hemodynamic, hematologic and metabolic outcomes by birth weight of preterm infants.

	AGA (N = 81)		SGA (N = 60)		p
**Surfactant:** ≥ 2 doses (n, %)	21	25.9	20	33.3	.34
**Pneumothorax** (n, %)	0	0.0	1	1.7	.43
**Pulmonary hemorrhage** (n, %)	12	14.8	10	16.7	.77
**Respiratory distress syndrome** (n, %)	48	59.3	37	61.7	.77
**HFO** (n, %)	22	27.2	27	45.0	**.03**
**Duration of endotracheal ventilation**^#^ (days) (median, IQR)	1.0	[0.2;6.0]	5.0	[0.5;14.4]	.09
**Duration of non-invasive ventilation**^#^ (days) (median, IQR)	30	[14;40]	37	[28;48]	**< .01**
**Age at final extubation** (days) (median, IQR) (for intubated newborns)	13	[1;23]	19	[8;29]	.09
**Treatment for pulmonary hypertension** (n, %)	5	6.2	10	16.7	**.05**
**Steroid therapy before 36 weeks’ gestation** (n, %)					
Systemic	3	3.7	2	3.3	.99
Inhaled	4	4.9	8	13.3	.08
**Inotropic drugs** (n, %)	21	25.9	25	41.7	**.05**
**Vascular filing** (n, %)	4	4.9	9	15.0	**.04**
**PDA** (n, %)	48	59.3	35	58.3	.91
**Surgical treatment of PDA** (n, %)	4	4.9	6	10.0	.33
**Insulin treatment** (n, %)	22	27.2	35	58.3	**< .001**
**Number of red blood cell transfusion** (median, IQR)	1	[0;2]	2	[1;4]	**< .001**
**Nadir platelets in the 1st week of birth** (x10^3^/mm^3^) (median, IQR)	102	[58;152]	70	[46;128]	**< .01**

^#^: Among infants alive at 36 weeks’ gestation. HFO: high-frequency oscillation. PDA: patent ductus arteriosus.

IQR: interquartile range.

Any missing data for each variable

### Primary and secondary outcomes

Overall, 61.5% (32/52) and 27.4% (20/73) of SGA and AGA newborns showed moderate or severe BPD (p < .001; Table 3). This difference remained significant after adjustment for GA (OR = 5.2, 95% CI [2.2–12.4]). In addition, the composite outcome death or moderate to severe BPD at 36 weeks’ PMA was more frequent for SGA than AGA newborns (66.7% vs 34.6%, p < .001), which persisted after adjustment for GA (OR = 4.1, 95% CI [1.9–9.1]). Death, severe neurological injury, retinopathy and necrotizing enterocolitis did not differ between SGA and AGA infants. Late-onset infections were more frequent in SGA than AGA infants (76.7% vs 53.1%, p<0.01, adjusted on GA OR = 2.8, 95% CI [1.3–6.1]).

**Table 3:** Primary and secondary neonatal outcomes by birth weight of preterm infants and association after adjustment on GA.

	AGA (N = 81)		SGA (N = 60)			Adjusted on GA		
	n	%	n	%	P	OR*	[95% CI]	P
**Moderate or severe BPD**^**a**^	20	27.4	32	61.5	**< .001**	5.2	[2.2–12.4]	**< .001**
**Death or moderate to severe BPD**	28	34.6	40	66.7	**< .001**	4.1	[1.9–9.1]	**< .001**
**Death**	8	9.9	12	20.0	.09	2.2	[0.8–5.8]	.13
**Severe neurological injury** (IVH grade III-IV, PVL)	10	12.3	2	3.3	.06	0.2	[0.1–1.1]	.06
**Retinopathy** stage 2 or 3 ^**a**^	2	2.8	0	0.0	.51	NA		
**Late-onset infections**	43	53.1	46	76.7	**< .01**	2.8	[1.3–6.1]	**.01**
**Necrotizing enterocolitis**	3	3.8	2	3.3	.99	0.9	[0.1–5.4]	.88

BPD: bronchopulmonary dysplasia. IVH: intraventricular hemorrhage. PMA: postmenstrual age.

PVL: periventricular leukomalacia.

*: odds ratio (OR) adjusted for GA. 95% CI: 95% confidence interval.

^**a:**^ (among 73 survivors in AGA and 52 survivors in SGA at 36 weeks’ PMA)

As compared with infants with no or mild BPD, those with moderate to severe BPD had lower median GA (27.6 vs 28.6 weeks, p < .001), lower median birth weight (690 vs 900 g, p < .001), higher lactate level at birth (4.8 vs 4.0 mmol/L, p < .01), and higher rate of PDA (73.1% vs 43.8%, p < .01) and more frequently received more than 2 doses of surfactant (44.2% vs 13.7%, p < .001). Male sex and antenatal corticosteroids use were not significantly associated with moderate or severe BPD in our population. However, because these variables are frequently described in the BPD literature, they were selected as potential confounding factors. On multivariate regression, risk of moderate to severe BPD was greater for SGA than AGA infants (OR = 5.9, 95% CI [2.2–15.4]), as was death or moderate to severe BPD (OR = 4.7, 95% CI [1.9–11.3]) (Table 4). Except for the dose number of exogenous surfactant, none of the other confounding factors (male sex, lactate level at birth, antenatal steroids and PDA) was significantly associated with the occurrence of BPD or the composite outcome death or moderate to severe BPD at 36 weeks’ PMA.

**Table 4:** Association between birth weight and moderate to severe bronchopulmonary dysplasia (BPD) and death or moderate to severe BPD at 36 weeks’ postmenstrual age (PMA).

	Moderate or severe BPD at 36 weeks’ PMA			Death or moderate to severe BPD at 36 weeks’ PMA		
	aOR*	95% CI	P	aOR*	95% CI	p
**Birth weight**			**< .001**			**< .001**
≥ 10^th^ percentile	1	-		1	-	
< 10^th^ percentile	5.9	[2.2–15.4]		4.7	[1.9–11.3]	
**GA** (weeks)	0.4	[0.3–0.7]	**< .01**	0.5	[0.3–0.7]	**< .001**

BPD: bronchopulmonary dysplasia. PMA: postmenstrual age. PDA: patent ductus arteriosus.

aOR: adjusted odds ratio. 95% CI: 95% confidence interval.

*: Adjusted on birth weight, gestational age, gender, lactate at birth, antenatal steroids, exogenous surfactant and patent ductus arteriosus.

A second analysis using birthweight z-score as a continuous variable and adjusted on the same potential confounding factors than the previous multivariate analysis was performed, and similar results were found: higher adjusted risk of moderate to severe BPD with decreasing birthweight z-score (OR = 0.25 [0.12–0.51], p < .001), and higher risk of death or moderate to severe BPD with decreasing birthweight z-score (OR = 0.32 [0.17–0.59], p < .01).

## Discussion

Among very preterm infants born between 24 and 30 weeks’ gestation to mothers with preeclampsia, SGA was associated with increased frequency of moderate or severe BPD and death or moderate to severe BPD at 36 weeks’ PMA.

The link between SGA and BPD has already been highlighted in several studies [2,4,5,17,18,19,20,21,22]. In the population-based cohort from Zeitlin *et al*., including 4525 infants born between 24 and 30 weeks’ gestation in nine European countries, BPD rate significantly increased with decreasing birth weight for GA; the adjusted OR for BPD was 6.4 with birth weight below the 10th percentile versus birth weight between the 50th and 74th percentile [2]. A French study in 2014 highlighted that a birth weight below the third percentile on AUDIPOG curves in infants born before 32 week’s gestation, is a stronger risk factor for BPD than extreme prematurity [21]. However, few studies have analyzed vascular diseases of pregnancy that may themselves be associated with BPD. Indeed, there is growing evidences that an appropriate angiogenic state is required for normal pulmonary vascular and alveolar development [10,11], and some studies found lower level of vascular endothelial growth factor (VEGF) in tracheal aspirates and higher level of soluble VEGF receptor (sVEGF-R or sFlt-1) in preterm infants with than without BPD [23]. Tang *et al*. showed that injection of sFlt-1 in amniotic fluid impaired the lung growth of rat pups [24]. Preeclampsia, as an anti-angiogenic state, might impair lung angiogenesis and lead to BPD, however, the link between preeclampsia and BPD is still not fully understood.

To our knowledge, studies about mortality and morbidity after premature birth often include together several contexts of premature birth or include preeclampsia in vascular disorders of pregnancy without studying separately preeclampsia. Only few studies specifically analyzed neonatal outcomes of newborns born to mothers with preeclampsia. None studied the outcome BPD at 36 weeks’ PMA. One study found increased risk of fetal and neonatal mortality with birth weight below the 10^th^ percentile but no differences for the other neonatal outcomes [12]. A second study found no significant differences between SGA and AGA infants, but infants born before 28 weeks’ gestation were excluded and the outcome was defined only as "death or need for neonatal intensive care" [13]. Some cohort studies have analyzed the incidence of BPD in different contexts of preterm birth. Eriksson *et al*., in a large retrospective cohort study in Sweden, including preterm infants born before 37 weeks’ gestation, found a strong association between preeclampsia-related disorders and BPD but did not adjust the analyses for GA [25]. An Italian cohort of 2085 infants born between 23 and 31 weeks’ gestation highlighted greater risk of BPD for those born during pregnancies with disorders of placentation than in a context of infection or inflammation [26]. However, none analysis of these studies were adjusted for birth weight. Overall, few studies approached the risk of BPD in SGA considering vascular disorders of pregnancy, and specifically preeclampsia. Otherwise, results often differ according to the studies. The risk of BPD was higher for babies born after pregnancies with vascular disorders and birth weight below the 10^th^ percentile in the MOSAIC cohort study [2] but higher odds were also observed for SGA born to mothers without vascular disorders. In the study by Durrmeyer *et al*. [27], preterm infants born to mothers with vascular disease with a birthweight below the 3rd percentile were at higher risk of BPD. A prospective study published in 2010 by Hansen *et al*. found preeclampsia as a risk factor for DBP with an OR at 2.96 in a population of premature newborns with an average GA 29 week’s gestation [28], while Yen *et al*. in 2013 found a negative association between preeclampsia and the risk of developing DBP [29]. Lastly, Soliman *et al*. didn’t find any association between preeclampsia and BPD development in a Canadian population of premature less than 32 week’s gestation [30].

In our study, we focused on a homogenous population of neonates born to mothers with preeclampsia. Moreover, our population concerned more immature infants than those in several already published studies. Moderate or severe BPD and death or moderate to severe BPD were more frequent for SGA than AGA preterm infants and this difference persisted after adjustment for GA. As SGA is frequently the consequence of fetal growth restriction, this result is consistent with Torchin’s study which showed that vascular pregnancy disorders, such as preeclampsia, HELLP syndrome or eclampsia, were factors involved in BPD development only if associated with fetal growth restriction [31].

Besides the main result, we detailed the respiratory management in our population, which indicated more prevalent respiratory morbidity in SGA than AGA infants: the need for endotracheal ventilation including HFO and for non-invasive ventilation was more frequent, and final extubation occurred later. Also, use of inhaled corticosteroids was more frequent for SGA than AGA newborns, although the association was not significant. They were used only to allow weaning of invasive ventilatory support.

We conducted this study to help us distinguish the respective role of preeclampsia and small for gestational age in bronchopulmonary dysplasia. In this study, we confirm that low birth weight for GA is a major factor in BPD development. On the other hand, several studies focused on the role of anti-angiogenic factors in this disease, referring to the “vascular hypothesis of BPD”, which implies a modified balance between pro and anti-angiogenic factors leading to an impaired vascular and alveolar development [32,33]. However, being small for gestational age seems to have an important effect on pulmonary development, even in the specific population of neonates born to mothers with preeclampsia and thus exposed to the same anti-angiogenic state than their AGA peers. Indeed, most studies about angiogenic and anti-angiogenic factors found similar profiles between SGA and AGA infants [34,35]. Only one study, to our knowledge, found discordant angiogenic profiles between preeclampsia alone and preeclampsia with SGA [36]. But these three studies focussed on less premature infants than our study. Nevertheless, it is possible that even among mothers with preeclampsia, the impact of maternal disease is variable on the foetus, with increasing severity of preeclampsia/vascular impairment contributing to the SGA state. Thus, SGA infants of preeclamptic mothers could represent a spectrum of disease rather than a separate entity independent of preeclampsia.

Our study presents several limitations: it was a retrospective, single-center cohort. The small sample size reduced the statistical power. We used birth weight as a proxy to identify growth-restricted preterm newborns. BPD status and severity were defined at 36 weeks PMA according to the National Institute of Child Health and Human Development definition [1], which has been shown to be associated with increased mortality and respiratory morbidity during infancy [37]. Therefore, administered respiratory support at 36 weeks PMA was used to classify children. The use of an oxygen reduction test would probably have been better to assess BPD status, but we don’t perform this test routinely in our unit, so we were not able to use that definition retrospectively [38]. In a recent systematic review of all papers published from 2010 and 2015 reporting BPD as an outcome, together with studies that compared BPD definitions between 1978 and 2015, 30% used the NICHD consensus definition and 6% used a physiological definition such as an oxygen challenge test [39]. However, we studied a very homogeneous population of premature infants, and management practices were the same for all newborns. Data collection was almost exhaustive. We analyzed two main outcomes, BPD and death or moderate to severe BPD, to consider a potential competitive effect of these two events and found greater risk of both outcomes for SGA than AGA newborns. Finally, our results agree with previous studies, showing more prevalent respiratory morbidities for SGA than AGA preterm infants.

In conclusion, in a homogeneous population of babies born to mothers with preeclampsia, we highlight the importance of birth weight for GA in the development of BPD. SGA in a context of preeclampsia multiplied by 5.9 in extremely preterm babies the risk of BPD development. These results encourage us to target this population for futures therapeutics studies. Indeed, SGA preterm infants are often excluded in therapeutics studies because of increased risk of complications or deaths in this population of premature children [40]. At present, these results can’t help clinicians in the absence of new therapies tested on this particular population.

## Abbreviations

AGA: appropriate for gestational age
BPD: bronchopulmonary dysplasia
CI: confidence interval
CRIB: Clinical Risk Index for Babies
GA: gestational age
HELLP syndrome: Hemolysis, Elevated Liver enzymes, Low Platelet count syndrome
HFO: high frequency oscillation
IQR: interquartile range
OR: odds ratio
PDA: patent ductus arteriosus
PMA: postmenstrual age
SGA: small for gestational age
VEGF: vascular endothelial growth factor
